# The Epidemiology of Aggression and Associated Factors among Iranian Adult Population: A National Survey

**DOI:** 10.34172/jrhs.2020.34

**Published:** 2020-11-25

**Authors:** Jalal Poorolajal, Bahram Ebrahimi, Forouzan Rezapur-Shahkolai, Amin Doosti-Irani, Mahnaz Alizadeh, Jamal Ahmadpoor, Leila Moradi, Azam Biderafsh, Fateme Nikbakht, Zakie Golmohammadi, Ehsan Sarbazi, Samira Bahadivand, Marzieh Jahani Sayad Noveiri, Maryam Rezaei, Somayeh Ghorbani Gholiabad, Saber Heidari, Hadi Bagheri, Mojtaba Ghalandari, Fatemeh Zeynab Kiani, Narges Fakhranirad, Saeed Ghavi, Parivash Seydkhani

**Affiliations:** ^1^Department of Epidemiology, School of Public Health, Hamadan University of Medical Sciences, Hamadan, Iran; ^2^Research Center for Health Sciences, Hamadan University of Medical Sciences, Hamadan, Iran; ^3^Social Determinants of Health Research Center, Hamadan University of Medical Sciences, Hamadan, Iran; ^4^Department of Public Health, School of Public Health, Hamadan University of Medical Sciences, Hamadan, Iran; ^5^Modeling of Noncommunicable Diseases Research Center, Hamadan University of Medical Sciences, Hamadan, Iran; ^6^Department of Medical Library and Information Sciences, School of Paramedicine, Hamadan University of Medical Sciences, Hamadan, Iran; ^7^Research Center for Environmental Determinants of Health, Kermanshah University of Medical Sciences, Kermanshah, Iran; ^8^Epidemiology and Biostatistics Department, Health Faculty, Tehran University of Medical Science, Tehran, Iran; ^9^Department of Epidemiology, School of Health, Mashhad University of Medical Sciences, Khorasan Razavi, Mashhad, Iran; ^10^Department of Midwifery, School of Nursing and Midwifery, Mashhad University of Medical Sciences, Mashhad Iran; ^11^Road Traffic Injury Research Center, Student Research Committee, Tabriz University of Medical Sciences, Tabriz, Iran; ^12^Student Research Committee, Hamadan University of Medical Sciences, Hamadan, Iran; ^13^Department of Biostatistics, School of Public Health, Hamadan University of Medical Sciences, Hamadan, Iran; ^14^Department of Epidemiology, School of Public Health, Kermanshah University of Medical Sciences, Kermanshah, Iran; ^15^Modeling in Health Research Center, Shahrekord University of Medical Sciences, Shahrekord, Iran; ^16^Department of Public Health, School of Public Health, Bojnurd University of Medical Sciences, Bojnurd, Iran; ^17^Departments of Epidemiology and Biostatistics, School of Public Health, Birjand University of Medical Sciences, Birjand, Iran; ^18^Department of Public Health, School of Public Health, Ilam University of Medical Sciences, Ilam, Iran

**Keywords:** Aggression, Adult, Risk factors, Behavior, Iran

## Abstract

**Background:** This survey was conducted to determine the level of aggression among the Iranian adult population and underlying predisposing factors.

**Study design:** A cross-sectional study.

**Methods:** This cross-sectional study included 10,957 participants, involving 23 out of the 31 provinces of Iran in 2019. The outcome of interest was aggression, evaluated by the Buss & Perry aggression questionnaire. The association between aggression and 34 demographic, behavioral, social, and cultural characteristics was assessed using simple and multiple linear regression.

**Results:** The overall mean (SD) score of aggression was 77.10 (22.53). Based on the severity of aggression, the participants were categorized into four groups as follows: 2,464 (23.1%) nonaggressive, 4,692 (43.9%) mild, 3,071 (28.8%) moderate, and 454 (4.2%) severe aggressive. Aggression was more likely to occur in people with the following characteristics: younger ages, having several siblings, lower ranks of birth, having an intimate friend of the opposite sex, having an aggressive father/mother, history of parental divorce, interest in watching action/porn movies, listening to music, history of escape from home/school, using neuropsychiatric drugs, using illicit drugs, history of suicidal thoughts/attempt, and family conflict and hostility. Aggression was less likely to occur with the following characteristics: reading, regular physical exercise, the ability to control anger, regular prayer, adherence to avoid lying, respect to other people's rights, sexual satisfaction, and attachment to parents.

**Conclusions:** A majority of the population has some degree of aggression. Aggression is a multifactorial behavior corresponding with several demographical, social, cultural, and religious factors, some of which back to early childhood events.

## Introduction

Aggression is overt or covert and can be defined in general terms as feelings of anger associated with hostile or violent behaviors that may result in harm or injure another person ^1,2^. Human aggression can be subdivided into direct and indirect. Direct aggression is characterized by physical or verbal behaviors and is associated to cause harm to someone. Indirect aggression is characterized by behaviors that may cause harm to the social relations of an individual or group ^3^.

Each year, more than 1.3 million people lose their lives worldwide as a result of violence and aggressive behaviors including self-directed, interpersonal, and collective ^4^. Among people aged 15 to 44 years, violence and aggressive behavior is the fourth leading cause of death globally ^5^. For every person who dies as a result of violence, there are many more people injured and suffer from a wide variety of physical, psychological, and sexual problems due to nonfatal aggressive behaviors ^6^.

There may be a link between different types of aggression and many underlying risk factors. For example, economic inequality ^4,7^, religion ^8^, marital status ^9^, media ^10^, prior punishment and child abuse ^11^, alcohol abuse ^12^, substance abuse ^13^, and many other factors may increase or decrease the risk of aggressive behaviors.

Severe international sanctions have been imposed on the Iranian population for many years. The sanctions have been associated with economic pressures that can influence the families living, particularly those in low socioeconomic levels. In addition to economic pressures, economic inequality can increase the risk of aggressive behaviors and depressed feelings ^4,7,14^.

Despite the importance of aggression and its health and social consequences, a comprehensive population-based survey has not been conducted yet in Iran to address the prevalence of different types of aggressive behaviors and their predisposing factors. The only information comes from small and unrepresentative samples of college students ^15,16^, wrestlers ^17^, intimate partners ^18^, or a limited population ^19^. There is no comprehensive information addressing aggression among the Iranian adult population. A better understanding of factors playing a role in developing aggression is required to implement effective prevention measures. Until reliable information on aggression and associated factors is collected, it is impossible to design effective intervention strategies and implement preventive programs.

 In 2019, we conducted a national survey to determine the prevalence and types of aggression among the Iranian adult population and associated underlying factors that may play a role in aggressive behaviors.

## Methods

 The Ethics Committee of the Hamadan University of Medical Sciences approved the study (IR.UMSHA.REC.1397.808). This cross-sectional study was performed on a relatively large representative sample of the Iranian adult population involving 10,957 participants from 23 out of the 31 provinces of the country in 2019.

 This survey was conducted on both male and female Iranian adults aged 18 years or older, irrespective of ethnicity, language, and place of residence. People of other nationalities were excluded from the study. The people participated voluntarily and anonymously in the study. A random sample was selected from each province. To make the samples more similar to the general population the participants were selected from various parts of the city where people go there for their daily affairs such as banks, drug stores, bus or subway stations, libraries, universities, campus, restaurants, parks, mosques, department stores, gyms, healthcare centers, clinics, and hospitals.

For the sake of sample size calculation, we considered the results of a cross-sectional study ^15^. Accordingly, the mean (SD) level of aggression score among the general population in one province of Iran was reported as 78.57 (19.25). Accordingly, we arrived at a sample size of 384 for each province at a 95% significance level and 0.1 error level. The sample size was rounded up to 400 for simplicity for each province. Finally, 10,957 samples were gathered from 23 provinces.

 We took a random sample of around 400 from the capital city of small provinces (with less than 4 million population) and a sample of around 800 from the capital city of big provinces (with more than 4 million population) as follows: Ahvaz (803), Arak (398), Birjand (399), Bojnurd (399), Ghazvin (300), Gorgan (345), Hamadan (397), Ilam (388), Isfahan (401), Karaj (387), Kermanshah (397), Khorramabad (400), Mashhad (698), Rasht (391), Sanandaj (410), Sari (435), Shahrekord (390), Shiraz (814), Tabriz (377), Tehran (1,409), Urmia (379), Yasuj (419), Zanjan (221). Eight out of 31 provinces were not included in this survey due to executive limitations, because we could not find a colleague to collect data.

 For all those involved in data collection to act in a coordinated manner, we created a Telegram channel for data collectors. In this channel, we taught the members how to collect data and provided them the necessary guidance, and asked the members to share their experiences and executive problems. Therefore, they all act in a coherent and coordinated manner.

 The data were collected through a self-administered questionnaire including demographic characteristics such as age, gender (male, female), educational level (illiterate, school education, academic education), marital status (single, married, divorced/widowed), number of siblings, the rank of birth. In addition, several behavioral, social, cultural and religious potential factors were evaluated such as the number of hours reading per week, having an intimate friend of the same sex (no, yes), having a friend of the opposite sex (no, yes), the number of times doing regular physical exercise per week, father's behavior (reasonable, aggressive, careless), mother's behavior (reasonable, aggressive, careless), death of a parent during childhood (no, yes), parental divorce during childhood (no, yes), ability to control anger (no, yes), doing regular prayer (no, yes), adhere to avoid lying (no, yes), respect to other people's rights (no, yes), interest in action movies (no, yes), interest in porn movies (no, yes), interest in music (no, yes), being sexually abused during childhood (no, yes), history of scape from home (no, yes), history of scape from school (no, yes), being in kindergarten during childhood (no, yes), using neuropsychiatric drugs (no, yes), using illicit drugs in the past month (no, yes), using alcohol in the past month (no, yes), having extramarital sex in the past month (no, yes), history of suicidal thoughts in the past month (no, yes), history of suicide attempt in the past year (no, yes), and having sexual satisfaction (no, yes). The common behaviors that take place very often were questioned for the past month while the less frequent behaviors were questioned for the past year.

Moreover, "attachment to parents" (low, high) and "family conflict and hostility" (low, high) were evaluated using the Violence-Related Attitudes, Behaviors, and Influences Assessment Tools among Youths introduced by the Centers for Disease Control and Prevention (CDC) ^20^. The "attachment to parents" included four four-choice questionnaires (NO, no, yes, YES, on a scale of 1-4) with scores ranged from 1 to 16. The "family conflict and hostility" included three four-choice questionnaires (on a scale of 1-4) with a total score ranged from 1 to 12.

The outcome of interest was aggression among adults, evaluated using the Persian version of the Buss & Perry aggression questionnaire (AGQ) ^21,22^. AGQ evaluates four domains of aggression, including physical aggression (questions 1-9), verbal aggression (questions 10-14), anger (questions 15-21), and hostility (questions 22-29). AGQ included 29 five-choice questionnaires (on a scale of 1-5) with a total score ranged from 29 to 145. The total scores of 29-58 were considered nonaggression, 59-87 mild, 88-116 moderate, and 117-145 a severe aggression. The Cronbach's alpha coefficient was 0.9258 for 29-item AGQ; 0.8454 for the domain of physical aggression; 0.7659 for the domain of verbal aggression; 0.7890 for the domain of anger; and 0.8451 for the domain of hostility.

 The association between aggression and independent factors was evaluated using a simple and multiple linear regression model. To find out which multiple linear regression model was the best fit for the data, we performed a backward stepwise regression method. For this purpose, we started with the full model and then excluded one variable at a time and compared the reduced model with the full model to choose the best-fitting model using the likelihood ratio test. The data were analyzed at a significance level of 0.05 using Stata software, ver. 16 (StataCorp, TX, USA).

## Results

The participation rate of the invited people was 88.6%. Of 10,957 participants, 5,755 (52.9%) were females. The mean (SD) age of the participants was 33.00 (11.31) years, ranged from 18 to 90 years. The frequency distribution of the demographic, behavioral, social, and religious characteristics of the participants is given in Table 1.

 The overall mean (SD) score of aggression was 77.10 (22.53) ranged from 29 to 145. The mean (SD) score of physical aggression was 22.67 (7.99) ranged from 9 to 45; verbal aggression 14.10 (4.92) ranged from 5 to 25, anger 19.09 (6.50) ranged from 7 to 35, and hostility 21.23 (7.67) ranged from 8 to 40.

 Based on the severity of aggression, the participants were categorized into four groups: 2,464 (23.1%) nonaggressive, 4,692 (43.9%) mild, 3,071 (28.8%) moderate, and 454 (4.2%) severe aggressive. Moreover, 276 questionnaires were incomplete.

**Table 1:** The frequency distribution of the demographic, behavioral, social, and religious characteristics of the participants

**Continuous variables**	**Mean**	**SD**
Age (yr)	33.00	11.31
Number of siblings	4.10	2.41
Rank of birth	2.87	1.91
Times of doing regular physical exercise per week	0.83	1.67
**Categorical variables**	**Number**	**Percent**
Gender		
Female	5755	52.9
Male	5121	47.1
Marital status		
Single	4140	38.0
Married	6321	57.9
Divorced/Widow	446	4.1
Educational level		
Illiterate	199	1.8
School education	4634	42.8
Academic education	6002	55.4
Reading at least one hour per week		
No	2724	28.0
Yes	6994	72.0
Having an intimate friend of the same sex		
No	2136	19.7
Yes	8711	80.3
Having an intimate friend of the opposite sex		
No	7472	69.0
Yes	3351	31.0
Father's behavior		
Reasonable	7537	70.2
Aggressive	1919	17.9
Careless	1276	11.9
Mother's behavior		
Reasonable	8294	77.1
Aggressive	1232	11.5
Careless	1226	11.4
Death of a parent during childhood		
No	9533	88.0
Yes	1301	12.0
Parental divorce during childhood		
No	10299	95.0
Yes	538	4.96
Ability to control anger		
No	3251	30.0
Yes	7597	70.0
Regular prayer		
No	5575	51.9
Yes	5165	48.1
Adhere to avoid lying		
No	3945	36.4
Yes	6895	63.6
Respect other people's rights		
No	2060	19.0
Yes	8782	81.0
Interest in watching action movies		
No	6492	59.9
Yes	4344	40.1
Interest in watching porn movies		
No	8172	76.2
Yes	2546	23.8
Interest in listening to music		
No	2174	20.1
Yes	8659	79.9
Being sexually abused in childhood		
No	9899	92.0
Yes	855	8.0
History of escape from home		
No	9682	89.6
Yes	1129	10.4
History of escape from school		
No	8381	78.0
Yes	2367	22.0
Being in kindergarten in childhood		
No	7780	72.1
Yes	3004	27.9
Using neuropsychiatric drugs		
No	9653	89.6
Yes	1122	10.4
Using illicit drugs in the past month		
No	9675	89.7
Yes	1110	10.3
Drinking alcohol in the past month		
No	9023	83.3
Yes	1803	16.7
Having extramarital sex in the past month		
No	9752	90.2
Yes	1064	9.8
A history of suicidal thoughts in the past month		
No	9884	91.3
Yes	947	8.7
A history of suicide attempt in the past year		
No	10249	94.6
Yes	588	5.4
Having sexual satisfaction		
No	973	15.8
Yes	5178	84.2
Attachment to parents		
Low	5288	52.7
High	4737	47.3
Family conflict and hostility		
Low	6729	64.1
High	3764	35.9

**Figure 1:**
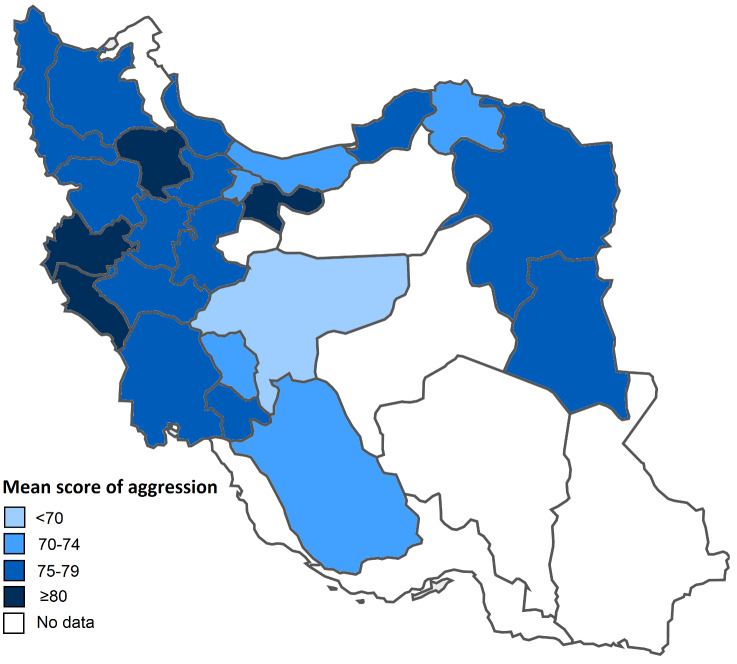
The provinces of Iran from which the samples were selected based on the severity of aggression

The mean score of aggression across provinces was statistically significant (*P*<0.001). The range of mean scores of aggression varied from 62.95 (in Isfahan) to 83.53 (in Zanjan). Accordingly, the 23 provinces were categorized into four groups with 5-score intervals as follows. The provinces with the mean score of <70 were denoted by the very light blue, the mean score of 70-74 by the light blue, the mean score of 75-79 by the blue, and the mean score of ≥80 by the dark blue (Figure 1).

Table 2 shows the associations between aggression and independent factors using a simple linear regression model. Based on this model, there was a significant association between aggression and almost all independent variables except for school education, the death of a parent during childhood, interest in music, and being in kindergarten during childhood.

Table 3 shows the associations between aggression and independent factors using a multiple linear regression model. According to the results of multiple linear regressions, there was a negative association between age and aggression. Therefore, the mean score of aggression decreased per each year increase in age. Both school and academic education reduced the level of aggression compared to illiterates. The level of regression was positively associated with the number of siblings. In other words, aggressive behaviors were higher among more crowded families. On the other hand, the level of aggression was negatively associated with the lower ranks of birth. That means the first offspring was more aggressive than the second one, the second one was more aggressive than the third one, and so on. The number of hours dedicated to reading per week was negatively associated with the level of aggression. The more someone read, the less aggressive they were. Moreover, doing regular physical exercise per week was negatively associated with the level of aggression. Having an intimate friend of the opposite sex had a positive relationship with the level of aggression. The level of aggression was higher among people whose fathers or mothers were aggressive than those whose fathers or mothers were reasonable. A history of parental divorce during childhood increased the level of aggression during adulthood. The ability to control anger was a characteristic that was strongly associated with a lower level of aggression. People who prayed regularly were less aggressive. The people who adhered to avoid lying and those who respected other people's rights were less aggressive. The interest in watching action and porn movies, as well as the interest in listening to music, was associated with an increased level of aggression. History of escape from home or school was strongly associated with an increased level of aggression during adulthood. Those who spent part of their childhood in kindergarten were less aggressive. Using neuropsychiatric drugs for medical reasons or other reasons, using illicit drugs, and drinking alcohol were associated with an increased level of aggression. There was a positive relationship between aggression and a history of suicidal thoughts in the past month or a history of suicide attempt in the past year. Attachment to parents reduced the level of aggression whiles family conflict and hostility were strongly associated with a higher level of aggression.

## Discussion

 In this national study, we found that aggression was a multifactorial behavior positively associated with several demographic, social, cultural, and religious factors, including younger ages, the number of siblings, lower ranks of birth, having an intimate friend of the opposite sex, having aggressive fathers or mothers, interest in watching action or porn movies, listening to music, history of escape from home or school, using neuropsychiatric drugs, using illicit drugs, history of suicidal thoughts or attempt, and family conflict and hostility. On the other hand, aggression was negatively associated with reading, regular physical exercise, having an intimate friend of the same sex, ability to control anger, regular prayer, adherence to avoid lying, respect for other people's rights, sexual satisfaction, and attachment to parents.

**Table 2:** The associations between aggression and independent factors using the simple linear regression model

**Variables**	**Const.**	**β**_1_	**SE**	**95% CI**		**t-test**	***P*** **-value**
Age (yr)	82.80	-0.17	0.02	-0.21	-0.13	-8.74	0.001
Gender (men vs. women)	75.07	4.30	0.43	3.44	5.15	9.87	0.001
Marital status (married vs. single)	79.42	-4.53	0.45	-5.42	-3.65	-10.04	0.001
Marital status (divorced/widowed vs. single)	79.42	7.70	1.14	5.46	9.94	6.75	0.001
Educational level (school education vs. illiterate)	81.91	-2.11	1.63	-5.31	1.09	-1.29	0.196
Educational level (academic education vs. illiterate)	81.91	-6.98	1.62	-10.17	-3.80	-4.30	0.001
Number of siblings	77.98	-0.21	0.09	-0.39	-0.03	-2.38	0.017
Rank of birth	78.47	-0.41	0.11	-0.65	-0.18	-3.52	0.001
Reading at least one hour per week (yes vs. no)	82.95	-8.01	0.51	-9.02	-7.01	-15.66	0.001
Number of times doing regular physical exercise per week	77.28	-0.57	0.14	-0.86	-0.28	-3.85	0.001
Having an intimate friend (yes vs. no)	80.05	-3.66	0.55	-4.74	-2.58	-6.66	0.001
Having an intimate friend opposite sex (yes vs. no)	74.16	9.43	0.46	8.51	10.34	20.28	0.001
Father behavior (aggressive vs. reasonable)	74.24	10.40	0.57	9.27	11.52	18.15	0.001
Father behavior (careless vs. reasonable)	74.24	9.32	0.67	8.00	10.64	13.83	0.001
Mother's behavior (aggressive vs. reasonable)	74.66	11.65	0.68	10.31	12.99	17.06	0.001
Mother's behavior (careless vs. reasonable)	74.66	10.01	0.68	8.67	11.34	14.70	0.001
Death of a parent during childhood (yes vs. no)	76.96	1.18	0.67	-0.14	2.50	1.74	0.081
Parental divorce during childhood (yes vs. no)	76.52	11.53	1.00	9.56	13.50	11.49	0.001
Ability to control anger (yes vs. no)	87.61	-14.97	0.45	-15.86	-14.07	-32.79	0.001
Regular prayer (yes vs. no)	82.24	-10.89	0.42	-11.73	-10.05	-25.42	0.001
Adhere to avoid lying (yes vs. no)	83.09	-9.41	0.44	-10.29	-8.54	-21.10	0.001
Respect to other people's rights (yes vs. no)	86.97	-12.16	0.54	-13.23	-11.08	-22.11	0.001
Interest in watching action movies (yes vs. no)	73.58	8.76	0.43	7.90	9.62	19.96	0.001
Interest in watching porn movies (yes vs. no)	74.10	12.59	0.50	11.60	13.58	25.04	0.001
Interest in listening to music (yes vs. no)	76.51	0.76	0.55	-0.31	1.84	1.38	0.167
Being sexually abused in childhood (yes vs. no)	76.13	12.60	0.80	11.03	14.18	15.69	0.001
History of scape from home (yes vs. no)	75.25	17.67	0.69	16.30	19.03	25.40	0.001
History of scape from school (yes vs. no)	74.12	13.75	0.51	12.74	14.75	26.83	0.001
Being in kindergarten in childhood (yes vs. no)	77.04	0.24	0.48	-0.71	1.20	0.50	0.617
Using neuropsychiatric drugs (yes vs. no)	75.68	13.56	0.70	12.18	14.93	19.29	0.001
Using illicit drugs in the past month (yes vs. no)	75.46	16.13	0.70	14.75	17.51	22.89	0.001
Drinking alcohol in the past month (yes vs. no)	74.74	14.28	0.57	13.16	15.40	25.00	0.001
Having extramarital sex in the past month (yes vs. no)	75.95	15.95	0.72	14.53	17.36	22.13	0.001
A history of suicidal thoughts in the past month (yes vs. no)	75.80	15.13	0.75	13.64	16.62	19.94	0.001
A history of suicide attempt in the past year (yes vs. no)	76.12	18.21	0.94	16.35	20.07	19.18	0.001
Having sexual satisfaction (yes vs. no)	84.91	-11.92	0.77	-13.44	-10.40	-15.38	0.001
Attachment to parents (high vs. low)	81.90	-9.66	0.44	-10.52	-8.78	-21.76	0.001
Family conflict and hostility (high vs. low)	71.91	14.38	0.44	13.51	15.24	32.63	0.001

**Table 3:** The associations between aggression and independent factors using the multiple linear regression model, adjusted for all variables in the table

**Variables**	**β**_1_	**SE**	**95% CI**		**t-test**	***P*** **-value**
Age (yr)	-0.12	0.02	-0.16	-0.09	-6.62	0.001
Educational level (school education vs. illiterate)	-0.68	1.38	-3.39	2.02	-0.50	0.620
Educational level (academic education vs. illiterate)	-2.71	1.41	-5.47	0.05	-1.92	0.054
Number of siblings	0.39	0.11	0.18	0.60	3.59	0.001
Rank of birth	-0.35	0.12	-0.59	-0.11	-2.84	0.005
Reading at least one hour per week (yes vs. no)	-2.54	0.44	-3.42	-1.67	-5.73	0.001
Number of times doing regular physical exercise per week	-0.40	0.11	-0.61	-0.18	-3.56	0.001
Having a friend opposite sex (yes vs. no)	2.10	0.45	1.21	2.99	4.64	0.001
Father behavior (aggressive vs. reasonable)	1.38	0.54	0.31	2.44	2.54	0.011
Father behavior (careless vs. reasonable)	0.99	0.63	-0.25	2.23	1.56	0.119
Mother's behavior (aggressive vs. reasonable)	2.90	0.63	1.67	4.13	4.63	0.001
Mother's behavior (careless vs. reasonable)	1.19	0.64	-0.07	2.45	1.86	0.063
Parental divorce during childhood (yes vs. no)	1.84	0.87	0.13	3.55	2.11	0.035
Ability to control anger (yes vs. no)	-8.19	0.43	-9.04	-7.34	-18.95	0.001
Regular prayer (yes vs. no)	-3.57	0.41	-4.38	-2.76	-8.64	0.001
Adhere to avoid lying (yes vs. no)	-1.54	0.44	-2.41	-0.68	-3.50	0.001
Respect to other people's rights (yes vs. no)	-1.96	0.56	-3.05	-0.87	-3.53	0.001
Interest in watching action movies (yes vs. no)	2.99	0.41	2.18	3.80	7.21	0.001
Interest in watching porn movies (yes vs. no)	3.08	0.49	2.12	4.04	6.28	0.001
Interest in listening to music (yes vs. no)	1.43	0.49	0.48	2.38	2.94	0.003
History of scape from home (yes vs. no)	3.34	0.71	1.95	4.72	4.73	0.001
History of scape from school (yes vs. no)	4.70	0.51	3.70	5.70	9.20	0.001
Being in kindergarten in childhood (yes vs. no)	-0.88	0.44	-1.75	-0.02	-2.00	0.046
Using neuropsychiatric drugs (yes vs. no)	4.41	0.65	3.14	5.68	6.82	0.001
Using illicit drugs in the past month (yes vs. no)	2.22	0.70	0.85	3.59	3.18	0.001
Drinking alcohol in the past month (yes vs. no)	1.03	0.59	-0.12	2.19	1.75	0.080
History of suicidal thoughts in the past month (yes vs. no)	2.09	0.74	0.65	3.54	2.84	0.005
History of suicide attempt in the past year (yes vs. no)	3.55	0.92	1.74	5.36	3.84	0.001
Attachment to parents (high vs. low)	-2.63	0.40	-3.41	-1.85	-6.65	0.001
Family conflict and hostility (high vs. low)	9.14	0.40	8.35	9.93	22.69	0.001
Constant	85.52	1.71	82.17	88.87	50.03	0.001

World Health Organization estimated 475,000 cases of homicide worldwide in 2012. Accordingly, the prevalence of fetal interpersonal violence was estimated at 6.7 per 100,000 population globally. The prevalence of homicide varies from 3.0-5.9 to more than 21.0 per 100,000 population. Based on this report, the prevalence of fetal interpersonal violence among the Iranian population was 4.8 (95% CI: 1.1, 21.0) which was classified among countries with low prevalence ^23^.

Our results indicated that the number of siblings and the lower ranks of birth had a positive association with the level of aggression. This finding was approved by another study, where a large family size might increase the rate of aggression among siblings ^24^.

Our findings revealed a positive relationship between fathers' or mothers' aggressive behaviors and aggression during adulthood. Moreover, family conflict and hostility were associated with a high level of aggression while attachment to parents was associated with a low level of aggression during adulthood. Harsh parenting increased the risk of aggression in offspring, whereas positive parenting protected against it ^24^. Parents can promote healthy behavioral development through warm relationships by fostering social values. Supporting parents and families can successfully reduce the risk of aggressive and violent behaviors ^25^.

The present study indicated that regular prayer was significantly associated with reduced levels of aggression. The effect of religion on aggression vary across communities and religions. Aggression decreased with industrialization in Protestant nations while increased with industrialization in non-Christian nations ^8^. In India, aggressive behaviors were less likely in Muslim than in Hindu ^26^.

Based on our results, there was a positive relationship between aggression and interest in watching action or porn movies and listening to music. Aggressive commercial movies could increase the risk of both physical and verbal aggression ^27^. Experimental studies conducted on both adults and children have shown that violent movies tend to escalate the latent aggression in viewers, often resulting in destructive actions of aggression ^28^. However, the relationship between aggression and music depends on the type of music. Listening to rap and heavy metal music and sexual-aggressive song can significantly increase not only the risk of aggressive behaviors but also the risk of risky behaviors such as drinking alcohol and using illicit drugs ^29-31^. On the other hand, listening to light music as well as group music interventions could reduce aggression and anxiety and improve self-esteem ^32-34^.

According to our findings, using illicit drugs and a history of suicidal thoughts or suicide attempts was significantly associated with increased levels of aggression. There is enough evidence suggesting illicit drugs, alcohol, smoking, educational levels are strongly correlated with physical aggression and self-harm such as suicide ^35-42^.

Our findings suggested that regular physical exercise per week can reduce the level of aggression. Moderate physical exercise is associated with less hostility and aggression and trait-anxiety ^43^. However, the type of physical exercise is important. An experimental study revealed that a significant reduction in aggressive feelings was only found in people exercised individually, but not in people who did combat exercise ^44^.

Based on our results, sexual satisfaction was associated with low levels of aggression. This finding was consistent with the results of previous studies. A study, evaluated the marital relations among former prisoners of war, reported that decreased marital satisfaction could increase verbal aggression ^45^. Another study, which assessed the relationship between relational aggression and sexual satisfaction, concluded that relational aggression can negatively influence sexual satisfaction; therefore, the lower the sexual satisfaction, the greater the perceived relational aggression ^46^.

Based on our findings, using neuropsychiatric drugs was associated with an increased risk of aggression. Psychological disorders increased the risk of aggression including self-directed or interpersonal aggressive behaviors^47-49^.

There were some limitations and potential biases in this study as follows. First, like any other cross-sectional study, this study was associated with an inherent bias because, in such observational studies, the exposure and the outcome are measured simultaneously. Therefore, cross-sectional studies cannot support causal inference because they have no time dimension. Furthermore, the relationship between exposure and outcome does not necessarily mean a causal relationship ^50^. Second, our data collection tool included some sensitive questions that are taboo culturally and religiously in Iran such as using illicit drugs, drinking alcohol, extramarital sex, and suicidal behaviors. Although participants filled out the questionnaires voluntarily and anonymously, it is still likely that some participants gave the incorrect answer to the sensitive questions. Therefore, these factors might be underestimated and the results might be biased. Third, the data were collected just from urban areas but not from rural areas due to executive problems. This may introduce selection bias to the results and limits the generalizability of the results.

 Besides these limitations and potential biases, this national survey included a large representative sample of the Iranian adult population from throughout the country involving 10,957 participants from 23 out of 31 provinces. Therefore, the body of evidence can reflect a clear picture of the prevalence and severity of aggression and associated factors among the general population in Iran.

## Conclusion

 More than 70% of the participants had mild to moderate aggressive behaviors based on the Buss & Perry aggression questionnaire. Aggression was a multifactorial behavior corresponding with several demographical, habitual, social, cultural, and religious factors, some of which back to early childhood events. However, this survey just included the Iranian urban population but not the rural areas. Therefore, the results of this survey cannot be attributed to the whole population as well as the rural area. Further investigations are required to estimate the level of aggression among the Iranian general population.

## Acknowledgements

 This was part of the MSc thesis in Epidemiology. We would like to appreciate the Modeling of Non-communicable Diseases Research Center and the Vice-Chancellor for Research and Technology of the Hamadan University of Medical Sciences for approval of this work.

## Conflict of interest

 The authors have no conflict of interest to declare.

## Funding

 The Vice-Chancellor of Research and Technology, Hamadan University of Medical Sciences funded this study (9711096777).

## Highlights

- The overall mean (SD) score of aggression was 77.10 (22.53).
- A majority of the Iranian urban adult population had mild to moderate aggressive behaviors.
- Aggression is associated with several demographical, habitual, social, cultural, and religious factors.
- Some predisposing factors of aggression back to early childhood events.
- This survey did not include the Iranian rural population.
